# The two-child limit for benefits in the Supreme Court: implications for public health

**DOI:** 10.1177/17579139211054117

**Published:** 2021-10-25

**Authors:** R Machin

**Affiliations:** Senior Lecturer in Social Work and Health, Social Work, Care and Community, School of Social Sciences, Nottingham Trent University, Room: 3218 Chaucer Building, 50 Shakespeare Street, Nottingham NG1 4FQ, UK

*In this article, Machin demonstrates how welfare provision is a key determinant of population health and the clear evidence to demonstrate that welfare reform has led to income insecurity and a wide range of health issues. This article considers the impact of the two-child limit on health and well-being, the consequences of the Supreme Court decision and emphasises the importance of social security as a significant public health concern*.

**Figure:**
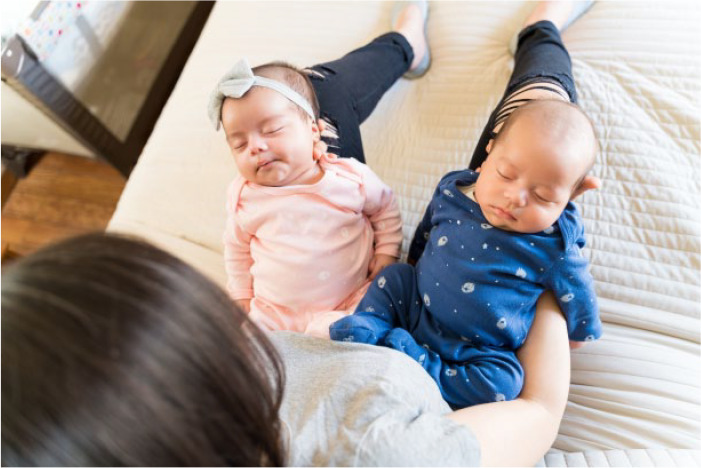


The last 10 years has been a remarkable period for the UK social security system. The most significant changes in welfare provision since the inception of the welfare state have resulted in a reduction in social security spending of over £30 billion. One of the most controversial elements of the government’s programme of welfare reform is the two-child limit. This policy has returned to the fore following a recent Supreme Court judgement,^1^ where the Court was asked to decide if the financial restrictions imposed by the two-child limit are compatible with rights under the European Convention on Human Rights. This commentary analyses the impact of the two-child limit, the consequences of the Supreme Court decision, and the ways in which social security is a significant public health concern.

The two-child limit was introduced from April 2017, and the child elements of Universal Credit and Child Tax Credit are no longer paid for the third or subsequent child in a family born after this date. The latest government figures^2^ show that 250,000 households and 911,000 children are affected by this policy and the numbers are increasing. The Institute for Fiscal Studies^3^ estimates that by the mid-2030s, 700,000 families will be impacted by the policy with an average reduction in income of £3000 per year; child poverty is projected to increase by 300,000. The government maintains that the two-child limit creates equity between working families and welfare recipients, although 57% of claimants affected by the policy are classified as being in-work.

The two-child limit creates a significant number of health and wellbeing concerns including an increase in mental health problems, strained relationships, social isolation, and guilt about an inability to support children in a family.^4^ The impact on children is stark; the social and educational development of younger children can be impeded, and older children can be excluded from social activities. There are a number of exceptions to the policy, including when a third child is born as part of a multiple birth, or adopted. The most controversial exception is based on ‘non-consensual conception’. Concerns have been expressed around the trauma of disclosing sexual violence, and the need to demonstrate that the claimant is no longer living with the perpetrator.^5^ Health and social care professionals can find themselves in the unwelcome position of acting as a ‘gate-keeper’ as third-party evidence must be provided to the Department for Work and Pensions confirming that a claimant’s circumstances are consistent with this exception. The British Pregnancy Advisory Service (BPAS)^6^ reports that the two-child limit was a key factor in many women deciding to terminate a pregnancy during the pandemic. It has been highlighted that the policy has a disproportionate impact on women, refugees, and families with larger families due to religious conviction or cultural norms.^7^ No other European welfare state has adopted a policy of the same nature as the two-child limit, and there have been calls for its abolition from the Children’s Commissioners of the devolved nations,^8^ and prominent public health professionals.^9^

A decision in the Supreme Court case, *SC and Ors v SSWP* UKSC 2019/0135, was handed down on 9 July 2021. The first claimant has a range of health problems which make the use of the contraceptive pill problematic; her third child was unplanned. The second mother fell pregnant with her third child after leaving an abusive relationship. Both women objected to abortion on moral grounds. The case, brought by Child Poverty Action Group, argued that women, large families, people with a moral or religious objection to birth control, children, and children with multiple siblings are unlawfully discriminated against as a result of the two-child limit. It was contended that this is a breach of Article 8 of the European Convention on Human Rights (right to respect for private and family life), Article 14 (prohibition of discrimination), and Protocol 1 of Article 1 (peaceful enjoyment of possessions). In a decision which disappointed many charities and social welfare lawyers, the Supreme Court held that the policy does not discriminate against women who are disproportionately affected by any policy linked to the raising of the children. It was judged that the government is entitled to administer a policy which it believes maintains economic wellbeing. In relation to larger families, the Court maintained that it should not interfere with government policy which seeks to balance state support provided to families and parental responsibility. The Court rejected that the two-child policy discriminates against children stating that the benefits system continues to support third or subsequent children through schemes such as child benefit and free school meals, and that children have no direct entitlement to welfare benefits as Universal Credit and Child Tax Credit are paid to adult carers.

The decision of the Supreme Court to approve the underlying principles of the two-child limit is not welcome news for low-income families or for public health. We know that as the gaps in the welfare safety net become wider, income and health inequalities increase. Craig and Katikireddi emphasise that welfare provision is a key determinant of population health.^10^ The Marmot Review 10 Years On^11^ highlighted that welfare reform has led to an increase in the number of families experiencing income insecurity; this is linked with adverse childhood experiences, poor long-term physical and mental health, and low life expectancy. Families experiencing financial insecurity have been more severely exposed to the health and economic impacts of COVID-19.^12^ The British Medical Association^13^ recognises that poverty alleviation should not be a marginal concern for health professionals. As the number of families affected by the two-child limit increases in the years ahead, it will be important for public health professionals to monitor how this policy undermines attempts to ‘level up’ inequalities.
